# Software Architecture for a Virtual Environment for Nano Scale Assembly (VENSA)

**DOI:** 10.6028/jres.109.018

**Published:** 2004-04-01

**Authors:** Yong-Gu Lee, Kevin W. Lyons, Shaw C. Feng

**Affiliations:** Gwangju Institute of Science and Technology, 1 Oryong-dong, Buk-gu, Gwangju, 500-712, Korea; National Institute of Standards and Technology, Gaithersburg, MD 20899-8263

**Keywords:** nanoscale assembly, software architecture, software reuse, VENSA, virtual reality

## Abstract

A Virtual Environment (VE) uses multiple computer-generated media to let a user experience situations that are temporally and spatially prohibiting. The information flow between the user and the VE is bidirectional and the user can influence the environment. The software development of a VE requires orchestrating multiple peripherals and computers in a synchronized way in real time. Although a multitude of useful software components for VEs exists, many of these are packaged within a complex framework and can not be used separately. In this paper, an architecture is presented which is designed to let multiple frameworks work together while being shielded from the application program. This architecture, which is called the Virtual Environment for Nano Scale Assembly (VENSA), has been constructed for interfacing with an optical tweezers instrument for nanotechnology development. However, this approach can be generalized for most virtual environments. Through the use of VENSA, the programmer can rely on existing solutions and concentrate more on the application software design.

## 1. Introduction

The two basic functions of a Virtual Environment (VE) development toolkit are managing different display devices (such as head-mounted displays, stereoscopic projection displays and haptic displays) and handling input devices (such as motion trackers, dials and buttons). In these toolkits, input and output devices are usually generalized by their similarities. For example, a magnetic position tracker and an optical position tracker have a common function, which can be generalized to a single class of position tracking devices. This allows the application programmer to write code using the generalized positional device without knowledge on which tracking device will be used [1]. Also, by defining the interfaces to these devices and always accessing the devices through these interfaces, the developed program becomes hardware independent. By constructing a development environment that can simulate this interface, one can develop and test programs on a host computer, and then run them on the actual device upon completion [2]. In addition to the above, VE toolkits provide many computer graphics and distributed computing techniques [3]. The latter is becoming more important for the following reason.

Designing and implementing the software for VE is becoming increasingly difficult as problem complexity grows and the expectation for presence realism increases. Fast computer processors are needed to achieve user requirements. This is typically achieved through proprietary parallel machines (high-end workstations) or through computer clusters (i.e., coordinated set of computers) interconnected by Fast Ethernet operating at 100 Mbit/s or Gigabit Ethernet operating at 1000 Mbit/s. Computer clusters are essential when the controllers to the peripherals can not all reside in a single computer. For example, some peripherals are based on a specific operating system or use a new interface standard, thus requiring another application specific computer to support it. Furthermore, computer clusters can be a good choice because they allow for incremental enhancement to the VE. New devices along with a new computer can be added without interfering with an existing computer cluster. With the rapid development of new input and output devices, it is becoming more certain that no one computer can meet the demands of future VE systems.

To achieve an immersive visual experience, one needs to provide from two to twelve visual displays. Two displays are needed for head-mounted displays and twelve displays are needed for six-walled screens such as CAVE^2^ (CAVE Automatic Virtual Environment) [4]. The graphics cards that generate these displays can reside in one proprietary computer or can be distributed within a computer cluster, and interconnected by a special network. Yet cluster programming introduces new issues such as synchronized management of distributed data and processes [5]. Furthermore the data from various input devices need to be propagated to other devices and systems and video retraces for the different video outputs must be synchronized [6].

Although VE programming is difficult, fortunately there are many software components, commercially available or in the public domain, that greatly reduce the development efforts. Some of the commercial toolkits are CAVELib [7] (www.vrco.com), WorldToolKit [8] (www.sense8.com) and DIVISION Reality [9] (www.ptc.com). Some of the public domain toolkits are VR Juggler [1], GNU/MAVERIK [3], MR Toolkit [10] and DIVERSE [11]. The first three support distributed programming^3^, with the first two offering companion toolkits. All of the toolkits provide fairly comprehensive functionality from low level device handling to sophisticated distributed process and data management.

Comprehensive VE toolkits are essential for rapid program development. Yet if a user wants to use only parts of several VE toolkits, implementing the VE becomes very difficult. This difficulty arises because most toolkits are frameworks that constrain the application programming to follow predefined rules. This makes it difficult to use a part without the whole.

VR Juggler, for example, completely manages the application program control by strictly defining the application functions that are called in predetermined order. The application program must provide the necessary functions that are executed through calls by the kernel. Some example functions in VR Juggler are preFrame(), intraFrame() and postFrame(). These functions are each called before, during and after the frame is refreshed.

MAVERIK, by contrast, is designed to have the data describing the application exist outside the framework. This is accomplished through its object manipulation structure called Shape Modeling Structure (SMS) [3] that uses callback functions to access the application data. Callback functions are provided by the application programmer. Although this approach separates the application data from the kernel, the callback pointers still put dependency of the application onto the MAVERIK.

Similar dependency problems exist for the following three VE toolkits. MR Toolkit uses Decoupled Simulation Model (DSM) [10] for structuring the components for the computation, presentation, interaction and geometric model. The interactions between these components are formally defined and the application program must follow rules defined by the DSM. DIVERSE heavily uses the scene graph functionality of Performer, a UNIX based graphics library for high-end visualization. DIVERSE uses DSO (Dynamic Shared Objects) to dynamically load executables in the UNIX [12] environment. This makes the toolkit limited to UNIX-based platforms that have Performer installed. VRPN [13] is attractive for users interested in solutions to small specific problems as it does not aim to provide an overall toolkit for VE. Rather, it focuses on the sub-problems of providing a uniform interface to various input and output devices.

This paper reports an effort at NIST (National Institute of Standards and Technology) to develop a VE for an optical tweezers system from concept to implementation. The main idea is to selectively use software components from existing VE toolkits without including their associated frameworks that can adversely influence the structure of the application model. This allows the application to be totally unaware of the VE toolkit. Distributed computing is achieved by duplicating the application on each computer. The purpose of distributed computing is to share the devices that are spread among three computers. Although this approach does not use the cluster resources optimally, it is well suited for the application described in this paper that does not require lengthy computations. For those that do, pre-computed lookup tables can be used.

## 2. Architecture

In this section, the background is introduced first, subsequently followed by the description of the hardware involved and the list of user requirements. Next the classes that fulfill the requirements are described using UML (Unified Modeling Language) [14]. Finally, problems that can result through the use of multiple VE toolkits are discussed.

### 2.1 Requirement Specification

This work is one part of a larger effort that has a goal of identifying and addressing fundamental measurement, control and standards issues related to manipulation and assembly of micro/nanoscale objects using optical methods. This developing system is called an Optical Tweezers (OT) and it uses a focused laser beam and a camera to move and track microscopic objects. Since the scale is too small for direct human manipulation, this effort defines a VE that will assist in manipulating, measuring and assembling nanoscale components.

**Fig. 1:**
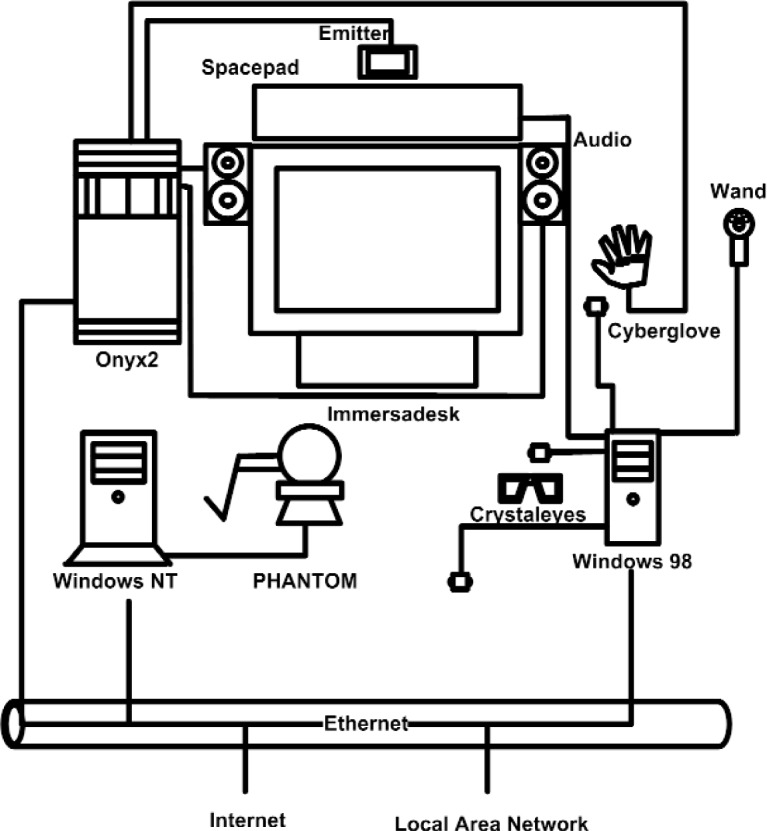
Hardware configuration of the virtual environment.

The hardware side of NIST’s VE consists of an Immersadesk [15] and a Cyberglove [16] controlled by an SGI Onyx2 workstation. The Spacepad [17] is controlled by a PC running Windows 98 operating system and a PHANTOM [18] is controlled by a PC running Windows NT operating system. Since all three computers use different operating systems we will refer to each computer by its operating system name. A schematic diagram is shown in Fig. 1. Onyx2 provides services for the audio, graphics and the Cyberglove. The stereoscopic vision is realized through the use of the large projection screen called an Immersadesk that achieves three-dimensional viewing through the use of special glasses called Crystaleyes. The Immersadesk is shown at the center and the Crystaleyes are shown left of the Windows 98. The video signal sync between the Immersadesk and Crystaleyes is achieved through an infrared emitter connected to the Onyx2. The Cyberglove, a device that tracks hand gestures, is shown to the right of Immersadesk. The Cyberglove, at the time of writing, was not incorporated into the architecture. Windows 98 administers the Spacepad, a device composed of one magnetic field generator (shown on top of the screen as a wide rectangle) and three receivers (small boxes coming out from the Windows 98). The Spacepad is used to track the movement of the head and the two hands. The Spacepad also provides a wand (small handle to the right of the Windows 98) composed of one dial and three buttons for issuing simple commands. Lastly, Windows NT is linked to the PHANTOM haptic device. All three computers communicate through the Ethernet.

**Fig. 2:**
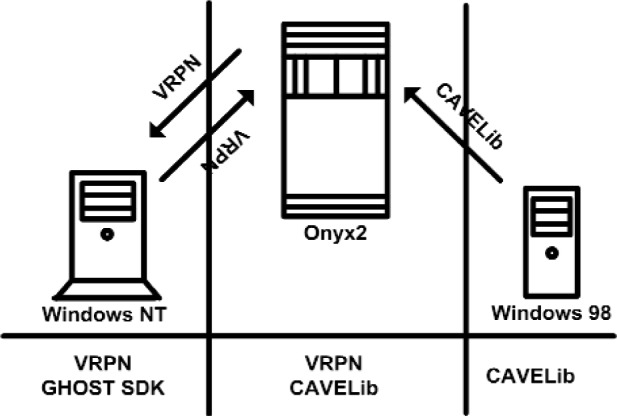
External virtual environment toolkits employed.

Next we shift our focus to the software components. Figure 2 illustrates the data flow between the computers and the VE toolkits that each use for controlling the devices. The toolkits involved in the data transmission are labeled above the arrows and they serve dual purposes, first for interfacing with the devices and second for the distributed computing. The detail is as follows. The tracking information gathered by the Windows 98 is sent to the Onyx2 by CAVELib. Onyx2 then relays it to Windows NT by VRPN. Similarly, Windows NT collects PHANTOM stylus position and orientation and sends it to Onyx2 by VRPN. Essentially, Onyx2 acts as the central input data collector and all collected data are propagated to the computer that requires it. In addition to CAVELib and VRPN, GHOST SDK is used to control the haptic device. GHOST SDK [19] is a commercial toolkit specialized for the PHANTOM haptic device and it uses a framework with a scene graph similar to OpenInventor. The use of multiple toolkits was required to meet the demands of the new system as the functionality that a single toolkit provided was not comprehensive. For example, VRPN has a native handling of the PHANTOM haptic device that the CAVELib lacks. GHOST SDK was later introduced because more control of the PHANTOM haptic device was needed than what VRPN could provide.

The VE is called VENSA (Virtual Environment for Nano Scale Assembly). The VENSA serves two purposes for the OT. The first is a simulation environment for nanoparticle interaction, and the second is an intuitive user interface for nanoassembly. Various meetings and interviews with optics, control and computer engineers led to the use case diagram illustrated in Fig. 3. All use cases and class diagrams used in this paper follow the convention of UML. Though not shown in this article, all class attributes and messages are modeled with UML and converted to C++ for implementation. The use of the diagram proved to be an efficient way of formalizing the processes that the engineers had in mind. After several iterations of feedback from the engineers and corresponding modification, the diagram was completed. The diagram consists of the whole process involved in the OT and some steps go beyond the scope of this article. In Fig. 3 (a), steps labeled 1.x.x are preliminary setup procedures. Step 3 is the shutdown procedure. The main interests are on steps labeled 2.x in Fig. 3 (b), which describe steering the particle. This illustrates the process when the operator is tele-operating the OT. In step 2.5, command is sent to the OT and the result is received in step 2.6. When VENSA is used in the simulation mode, a simulator substitutes for the OT in steps 2.5 and 2.6. This paper is primarily focused on the simulation mode.

### 2.2 Class Diagram

**Fig. 4:**
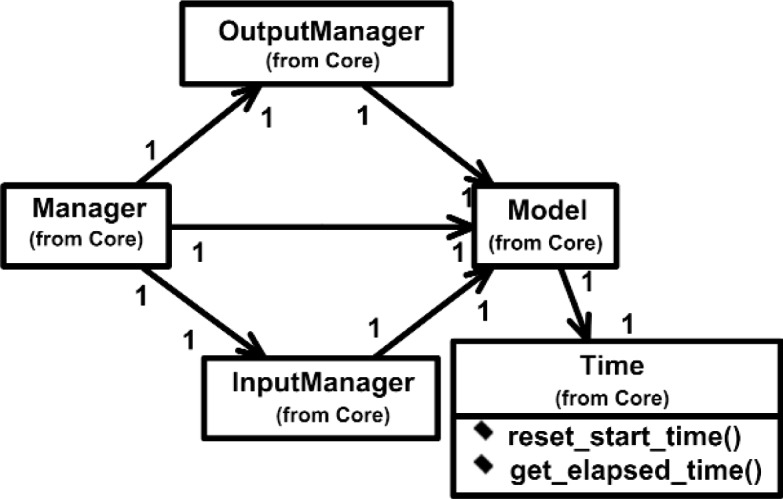
Relation between the Manager and the Model.

**Fig. 5:**
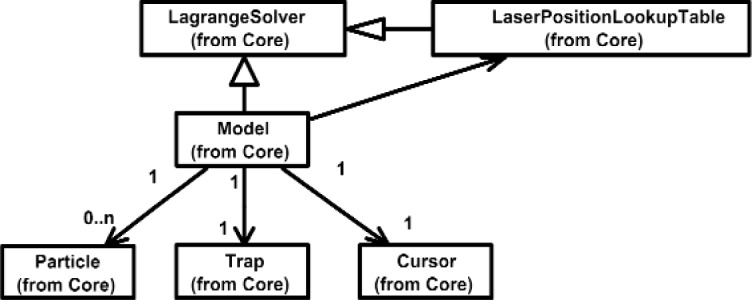
Model.

Overall, the Class Diagram for VENSA is illustrated in Figs. 4–8. When utilizing an Object-Oriented design process, it is a common practice to draw a sequence diagram for each use case. In doing so, objects and the messages that are sent between objects are defined. This is useful for process-intensive applications, yet for the application described in this paper, this stage was skipped going directly to the class diagram. Class diagram-to-usecase conformance was checked throughout the design process to verify that the classes were sufficient to implement the use cases.

Designed to be modular and extensible, the VENSA can be described by two important concepts, *functionality* and *generality*. The architecture functionally divides itself into *Model*, *Input*, *Output* and *Manager*. In Fig. 4, the Model is time-dependent central data that is modified by the *InputManager* as a result of various Inputs. Similarly, *OutputManager* modifies the Model and the various Outputs. The Model is modified twice within one cycle of the control loop, once first by the InputManager and subsequently by the OutputManager. The InputManager sets the initial condition of the model such as the initial position of the object to be moved. After that the control is passed to the OutputManager where the Model is aged according to the cycle time. The *Time* class shown in Fig. 4 calculates the cycle time by measuring the time lapse from the time when the program execution leaves InputManager to the next iteration. The messages involved in resetting the timer clock and obtaining the elapsed time are shown in the figure. Since it is impossible to know what the future cycle time will be, the previous cycle time is used as an approximation. Each InputManager and OutputManager can alter the Model but it must guarantee its integrity upon completion. Manager is the central supervisor for InputManager and OutputManager called *Manager*.

The architecture is also described in terms of its generality. Specific hardware devices are categorized under outer *Crust* and generalized devices that gather the commonalities among sets of similar devices are categorized as the inner *Core*. The class diagrams in Figs. 4 through Fig. 8 show this category in parenthesis. Core also includes all Model and Managers. Generality of the software functioning in Core enables the software to be extensible since new devices can reuse the Core through inheritance.

The Model is composed of *Particle*, *Trap* and *Cursor* as illustrated in Fig. 5. The Particle is the micro-to-nanometer scale object that is manipulated in VENSA. The external force that moves the particle comes from the Trap. The Cursor is a handle that is connected to the input device. For the OT application, the stylus of the PHANTOM haptic device is used as the input device. Notice that Model is a descendent of *LagrangeSolver*, the simulation engine of VENSA. Due to the computational overhead imposed by the LagrangeSolver, it is very difficult to retain the real time response. This is why a cached table called *LaserPositionLookupTable* is pre-computed before the simulation and used instead.

The Particle in Fig. 6 (a) is a *Molecule* or a *Continuum*. The geometry of the Continuum is modeled by the well known Constructive Solid Geometry (CSG) [20] or Boundary Representation (Brep) [20]. It has an attribute to represent its *materialType* (not shown). Molecule is simply a collection of *Atoms*.

A Trap such as shown in Fig. 6 (b) can have multiple *Potentials*. A Potential is a spatial function representing the potential energy of the particle placed in the potential field. Potentials can be created through dithering of the laser beam (e.g., time sharing one laser) or through splitting the single laser beam into multiple beams.

The Inputs shown in Fig 7 are designed through the use of inheritance [21]. For example in Fig. 7 (b), the common functions and attributes of *InputButtonSpacepad*, *InputButtonKeyboard*, *InputButtonGhost* and *InputButtonMouse* are moved up to *InputButton*. Again, the common functions and attributes of Fig. 7 (b) through Fig. 7 (f) are moved up to Input shown in Fig. 7 (a). The note boxes attached to the classes are names of the computers where the classes are implemented. Details will be discussed later. Notice the class hierarchy is not driven by the physical appearance of the hardware device. For example, a mouse device has buttons and a track ball for sensing the planar motion. This device is represented by two separate classes, InputButtonMouse [Fig. 7 (b)] and InputPositionalMouse [Fig. 7 (e)]. The design of Outputs are similar to Inputs as inheritance is also extensively used for them. VENSA supports visualization through *OutputGraphical*, haptic through *OutputHaptic* and audio through *OutputAudio*. This is illustrated in Fig. 8 (a). In Fig. 7 and 8, the physical Inputs and Outputs are those at the leaves. Notes are attached to the leaf Inputs and Outputs indicating the physical computer to which the device is attached. The computer names used in Figs. 7 and 8 are IRIX, WIN98, NT and NTGLUT. NT and NTGLUT can be thought of as the same computer. They are differentiated to support two different windowing libraries. One is through the GHOST SDK and the other is through GLUT. GLUT [22] is the OpenGL Utility Toolkit, a window system independent toolkit for writing OpenGL programs. Some class names are not self-explanatory and need to be clarified. *InputButtonGhost* [Fig. 7 (b)] refers to the button attached to the PHANTOM stylus. *InputDialSpacepad* [Fig. 7 (c)] is a dial attached to the wand that is part of the SPACEPAD. *InputDirectional* [Fig. 7 (d)] refers to devices that output two-dimensional unit-sized directional vectors. The best example is a joystick. *InputPositionalGhost* [Fig. 7 (e)] is the six-dimensional position and orientation information of the stylus of the PHANTOM device. *InputSpeechDragonNaturallySpeaking* [Fig. 7 (f)] is a text string converted from speech input through microphone. The conversion is done by a speech recognition software called Dragon Naturally Speaking. This feature was not implemented at the time of this writing. *OutputGraphicalOpenGL* [Fig. 8 (b)] is the parent class of OpenGL based graphical output devices. The three subclasses of OutputGraphicalOpenGL implement windowing functions that are missing in the parent class. *OutputGhostGraphical* uses the GHOST SDK, *OutputGLUTGraphical* uses the GLUT library and lastly, *OutputIRIXGraphical* uses the X-Window system. The haptic device is handled by *OutputGhostHaptic* [Fig. 8 (c)], a descendent of OutputHaptic. Lastly, the sound device is controlled by the *OutputIRIXAudioLibrary* [Fig. 8 (d)], a descendent of OutputAudio.

### 2.3 Existing Software Conflicts

**Fig. 9:**
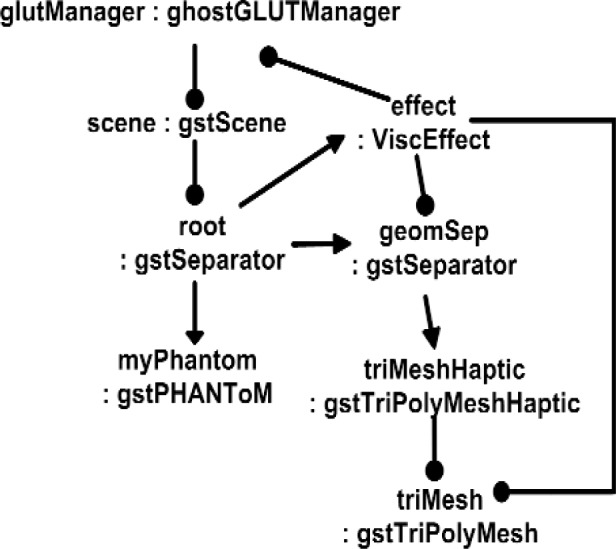
GHOST SDK scene graph.

Typical applications must interact with external libraries. Unfortunately, some libraries have their own architecture or class hierarchy that makes it very difficult to use them without abiding by the rules imposed by its associated framework. Interoperability is the most difficult problem in designing a new architecture. Some parts of the GHOST SDK for the PHANTOM haptic device shows this problem. Figure 9 illustrates the instantiation diagram of a sample GHOST SDK. The labels show instantiation names and class names separated by a colon. All classes are those of GHOST SDK. The circular ended arrows are the internal connections the programmer needs to explicitly set. The graph that connects the instances by triangular ended arrows is called *the scene graph*, a concept derived from OpenInventor. This also needs to be explicitly set. Notice these connections follow the programming rules of the GHOST SDK and the user must strictly follow these rules. The scene graph is built from the application data. When the application data changes, the scene graph needs to change accordingly. This requires that one maintain dual representations which can be problematic.

The scene graph is typically a part of most VE toolkits, thus making modularity difficult due to its a unique data structure. For efficiency, most applications have pointers to the scene graph from the data. Yet this causes the application program to become dependent on the scene graph. This work chose not to create any link between the application data and the external toolkit data. This provided support for modularity through some sacrifice in performance.

**Fig. 10:**
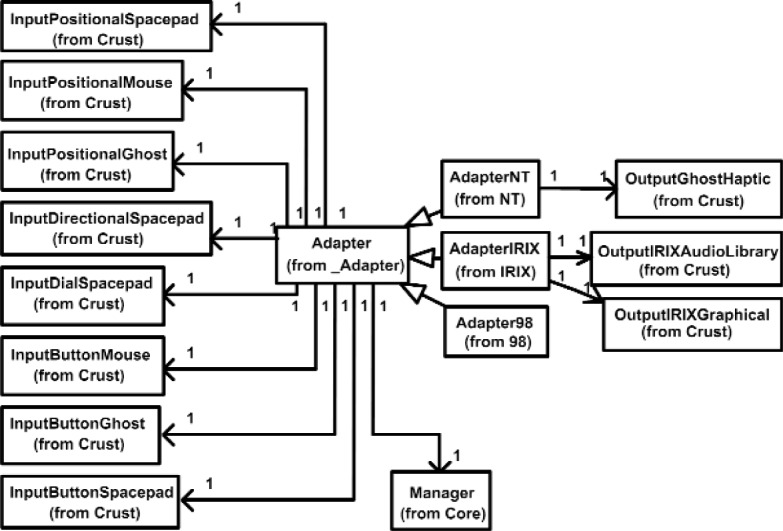
Adapter.

To maintain a clear modular architecture, it is not acceptable to have GHOST SDK objects or other framework objects in VENSA. This is enabled by using the *Adapter* that hides the complex GHOST SDK objects from the rest of the program. Adapter works as a wrapper to external libraries and relays the needed data flow to and from the VENSA objects. Adapter is also where the state of the Input and Output objects are realized to the physical hardware device. In Fig. 10, AdapterNT is the Adapter realized in the Windows NT. Similarly, there are AdapterIRIX and Adapter98 for the Onyx2 and Windows 98 platforms, respectively. Notice AdapterNT is the only one that has access to OutputGhostHaptic, since it is the only device that is physically connected to it. Similar rules apply to AdapterIRIX and Adapter98. Adapter98 does not have any output devices associated to it because it only serves as an input platform. Notice however all input devices are associated to all three Adapters (AdapterNT, AdapterIRIX, Adapter98). Physically, they are connected to only one platform, but the states of the devices are shared among all platforms. Note the VENSA architecture does not explicitly define the mechanism of sharing the device states among different platforms. The implementation detail is left to the user. This way all application programs located at each platform can work identically with the same input devices, making cluster programming simpler. In addition to device states sharing, the application program is duplicated among the platforms. This minimizes the amount of data that must travel through the network, thus requires little network bandwidth. A prior test implementation where the application program resided in one machine and the results were sent to the rest of the computers resulted in the failure of the haptic device, which requires continuous feeding of force vectors at 1 KHz.

### 2.4 Process Communications

There are two important processes in VENSA. First is the application process. This is where the time transient behavior of the model is computed. Second is the Adapter process that delivers the inputs from various input devices (sensors) to the application process and also conveys the resulting outputs to various output devices (such as visual, audio and haptic devices). This is illustrated in Fig. 11 (a). To the left is the main application cycle. The OutputManager and the InputManager constitutes the engine of the program. They compute the output device states from given input device states. InputManager computes the necessary state of the Model such that at the end of the cycle the Model would change to the determined state with the given Inputs and the cycle time. It then forwards the changed Model to the OutputManager. OutputManager then updates the Model and computes the output device states that reflect the new Model. The output device states are relayed to the necessary Output devices through Adapters. At the center is the Adapter cycle working as the bridge between application cycles and the input and output device cycles. Note all processes form a cycle and continuously run until program termination. The small looped cycles to the right and bottom of the Adapter cycle are the input and output device cycles. All adjacent cycles interchange Input and Output device states. The method of information flow is through polling. The receiver explicitly requests the sender for new data. In this way the sender never has to idly wait for sending out the new data and all processes actively run at all times. The example data flow is shown in Fig. 11 (b). For clarity only one input and one output cycle are shown. Steps 1 and 2 show how the input is transferred to the InputManager. Steps 3 and 4 show how the resulting output is transferred to the output device. In actual deployment, the Adapter cycles execute in each computer as in Fig. 11 (c). The communication between computers is done by star topology where a central computer (AdapterIRIX cycle) takes the role of relaying the information between the other two computers (Adapter98 and AdapterNT cycles).

In simulation mode, VENSA uses a constant time lapse. In future implementations where VENSA will be used as a tele-operation platform, the time delay before the computed output device setting will actually be relayed to the corresponding device needs to be predicted.

## 3. Discussion

One of the main objectives of VENSA is to keep the application program isolated from the complexity of the device handling. With VENSA much of the input and output device handling is done through the Adapter and the architecture leaves it as the implementers’ responsibility. Existing VE toolkits are sufficient to solve this implementation. Still the problem remains if one looks into how multiple VE toolkits would work in harmony within the Adapter. The practice chosen was to partition the internal code of Adapter such that only one VE toolkit resides in any one partition. And data duplication between different partitions was generously allowed. Of course this strategy was not the most efficient solution but it was thought that optimizing this problem was not worth the effort.

Since all the platforms supported OpenGL, the OutputManager generates all the graphics scenes optimally suited to OpenGL with codes that depend on OpenGL. This deviated from the philosophy of not depending on external toolkits. But since all graphics devices were based on OpenGL, it was not justifiable to define a new neutral graphics scene descriptor and have the output devices convert it to OpenGL primitives. However neutral data was used for the audio and haptics. Obviously they were much simpler to define than graphics.

This paper does not discuss using toolkits that provide advanced algorithms needed for VE, such as collision detection. These advanced toolkits differentiate themselves from the functions of the VE toolkits that are utilized in VENSA that are mostly device interfaces and device state propagation. These advanced toolkits require direct links to the application objects and sometimes may require some change in the application data structure. Certainly, combining the application program with these toolkits can destroy the modularity. The quick solution that we have implemented is to duplicate the application objects. One is used internally and the other is used for the specific advanced toolkits.

The application objects and input and output device objects are all static in VENSA. Dynamic object creation and destruction are not provided. For VENSA to be a dynamic environment, this functionality is essential. Note in cluster computers environment, all distributed applications must work coherently and must allocate or free objects as necessary.

## 4. Conclusion

Software reuse is important as it saves time and costs. By effectively reusing existing components, more effort can be put into problem solving. There exists a plethora of toolkits for VE today. However, no single toolkit could satisfy the needs of the application described in this paper. Multiple toolkits were needed to satisfy the needs. It is desired and beneficial to selectively choose certain features from a toolkit without the constraints associated with its framework. This is generally not possible as most toolkits are provided as part of a framework, which makes it very difficult, or impossible, to isolate a feature. Another approach is to use multiple toolkits together. However, using multiple toolkits requires that application code contain multiple interfacing codes to the toolkits, hence further complicating the modularity. Some toolkits are more problematic as they contain their own control loop and never return to the caller. The architecture of VENSA is designed such that it can incorporate existing VE toolkits without interfering with the application program code. Although VENSA runs on three VE toolkits, the VENSA classes show no dependence to on any of the toolkits. Any existing toolkits can be substituted for other toolkits without requiring the rewrite of the application code.

## Figures and Tables

**Fig 3 (a).**
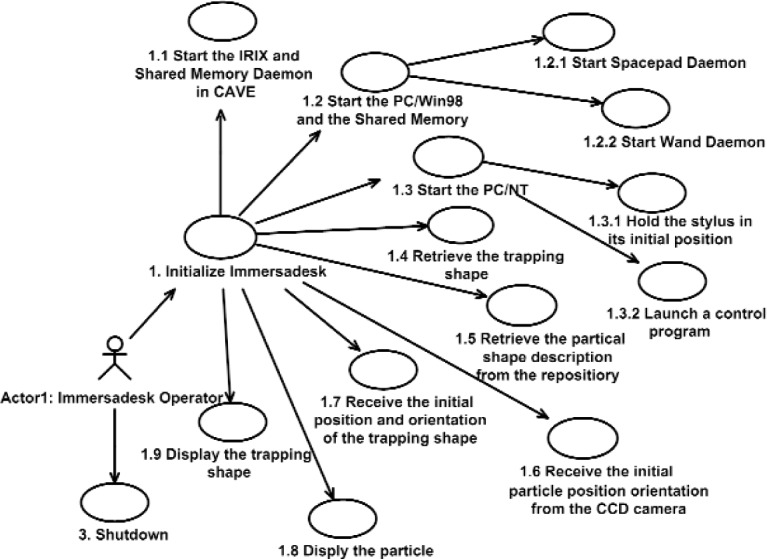
Use case diagram of VENSA. Initialization and shutdown.

**Fig 3 (b).**
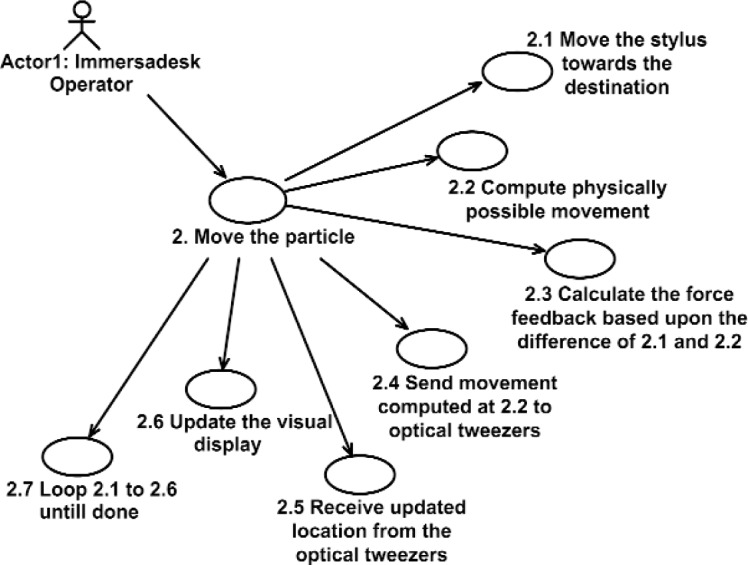
Use case diagram of VENSA. Particle manipulation.

**Fig. 6 (a):**
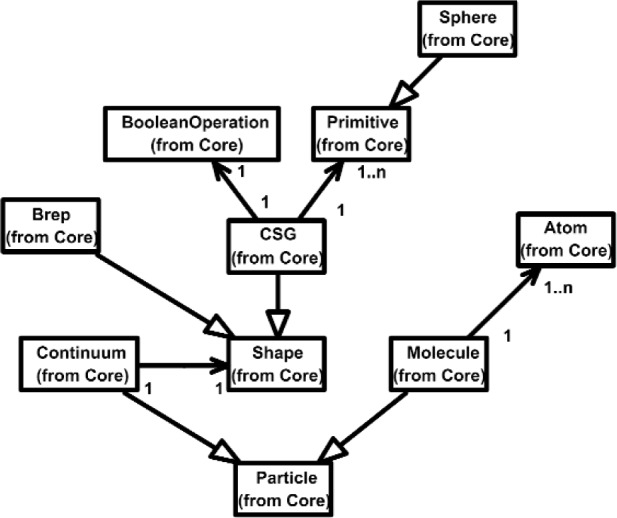
Particle of Model.

**Fig. 6 (b):**
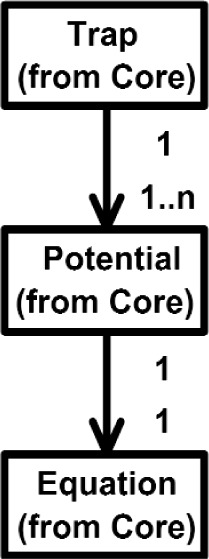
Trap of Model.

**Fig. 7 (a):**
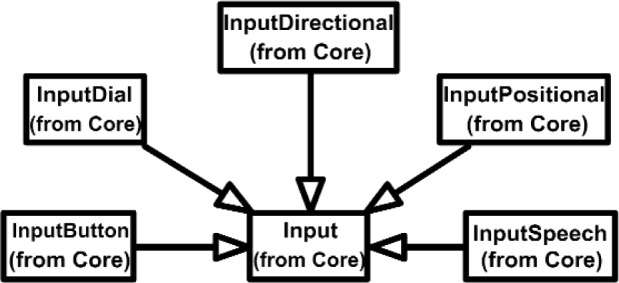
Overall view of Input components.

**Fig. 7 (b):**
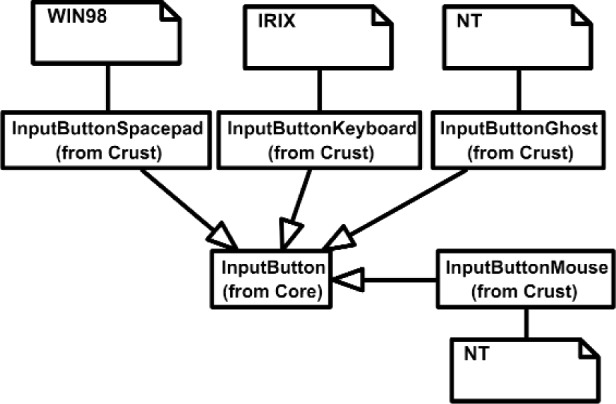
InputButton.

**Fig. 7 (c):**
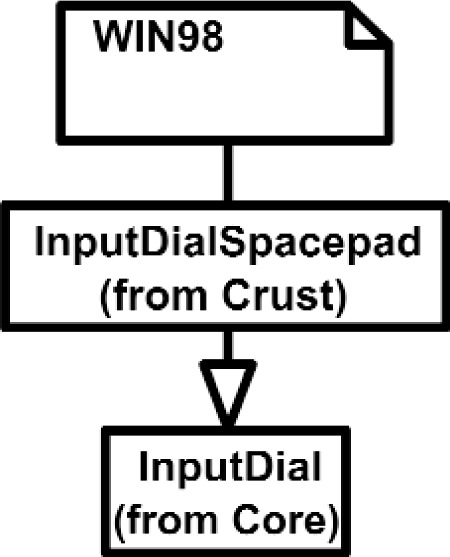
InputDial.

**Fig. 7 (d):**
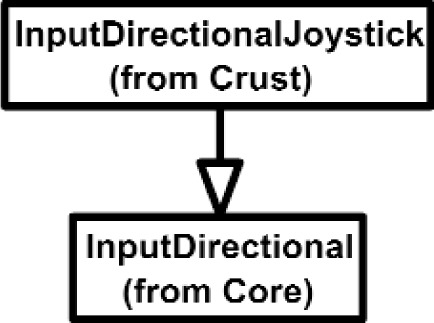
InputDirectional.

**Fig. 7 (e):**
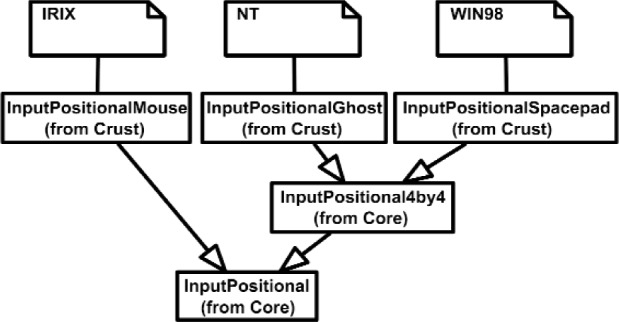
InputPositional.

**Fig. 7 (f):**
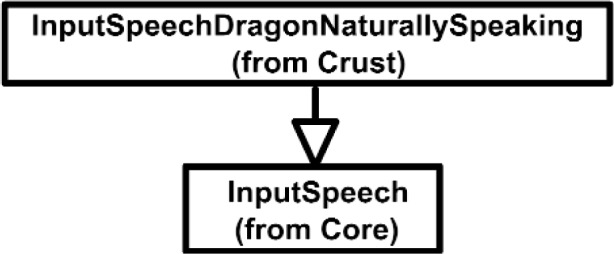
InputSpeech.

**Fig. 8 (a):**
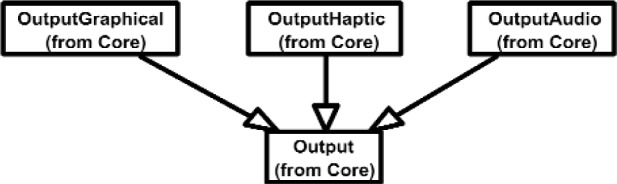
Overall view of Output components.

**Fig. 8 (b):**
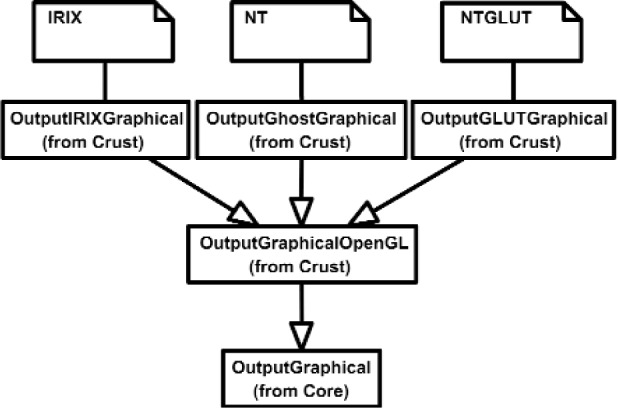
OutputGraphical.

**Fig. 8 (c):**
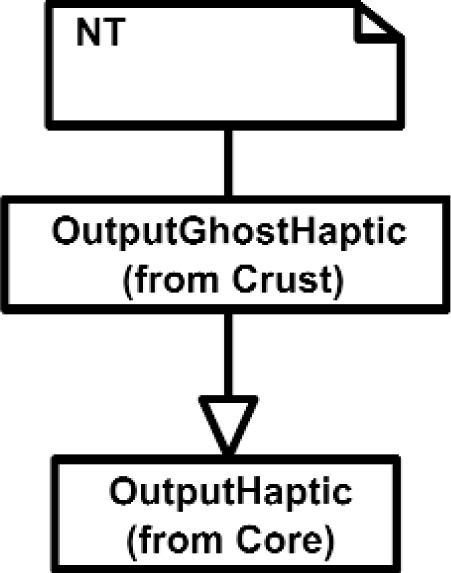
OutputHaptic.

**Fig. 8 (d):**
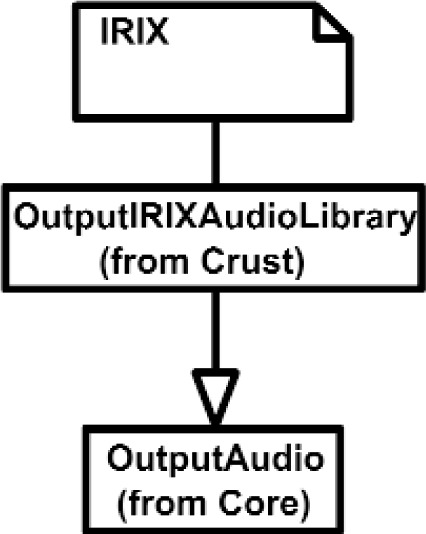
OutputAudio.

**Fig. 11 (a):**
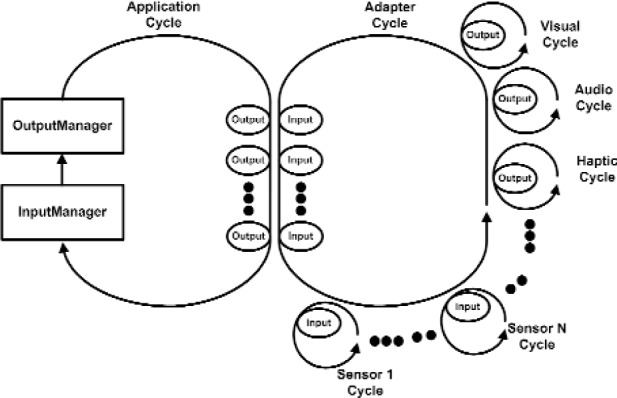
Relations between cycles within a platform.

**Fig. 11 (b):**
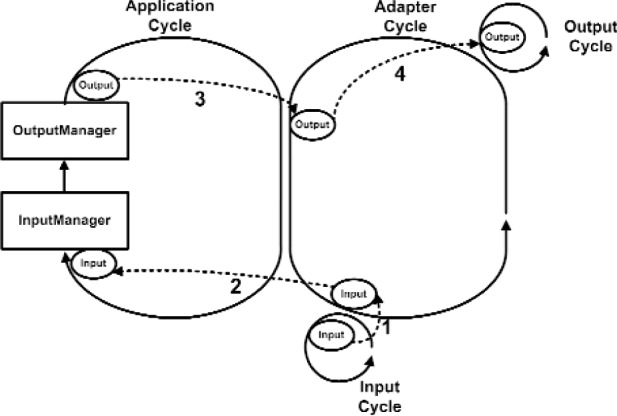
Information flow steps of Inputs and Outputs.

**Fig. 11 (c):**
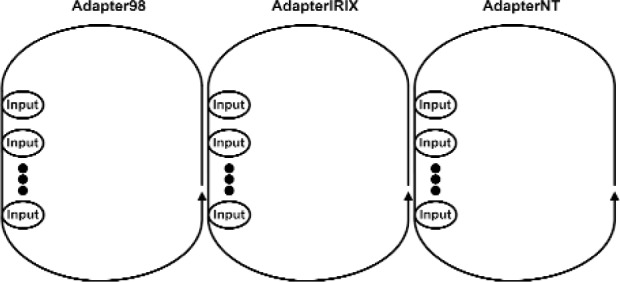
Relations between cycles across platforms.

## Footnotes

2 Certain commercial equipment, instruments, or materials are identified in this paper to foster understanding. Such identification does not imply recommendation or endorsement by the National Institute of Standards and Technology, nor does it imply that the materials or equipment identified are necessarily the best available for the purpose.

3 Cluster programming and distributed programming are terms used interchangeably throughout this paper.
